# Human cytomegalovirus microRNAs are carried by virions and dense bodies and are delivered to target cells

**DOI:** 10.1099/jgv.0.000736

**Published:** 2017-06-05

**Authors:** Abdul-Aleem Mohammad, Helena Costa, Natalia Landázuri, Weng-Onn Lui, Kjell Hultenby, Afsar Rahbar, Koon-Chu Yaiw, Cecilia Söderberg-Nauclér

**Affiliations:** ^1^​ Department of Medicine, Solna, Experimental Cardiovascular Unit, Department of Neurology, Center for Molecular Medicine, Karolinska Institute, Stockholm, Sweden; ^2^​ Department of Oncology-Pathology, Karolinska Institutet, Cancer Center Karolinska, Karolinska University Hospital, Stockholm, Sweden; ^3^​ Department of Laboratory Medicine, Karolinska Institute, Huddinge, Sweden

**Keywords:** HCMV miRNA, human cytomegalovirus, virions, dense bodies, lncRNA2.7

## Abstract

Human cytomegalovirus (HCMV) infection results in the production of virions, dense bodies (DBs) and non-infectious enveloped particles, all of which incorporate proteins and RNAs that can be transferred to host cells. Here, we investigated whether virions and DBs also carry microRNAs (miRNAs) and assessed their delivery and functionality in cells. Human lung fibroblasts (MRC-5) were infected with the HCMV strain AD169, and conditioned cell culture medium was collected and centrifuged. The pellets were treated with RNase-ONE, and the virions and DBs were purified with a potassium tartrate–glycerol gradient and dialysed. The virions and DBs were incubated with micrococcal nuclease, DNA and RNA were extracted and then analysed with TaqMan PCR assays, while the proteins were examined with Western blots. To assess the delivery of miRNAs to cells and their functionality, virions and DBs were irradiated with UV light. The purity of the virions and DBs was confirmed by typical morphology, the presence of the structural protein pp65 and the HCMV genome, the ability to infect MRC-5 cells and the absence of the host genome. RNA analysis revealed the presence of 14 HCMV-encoded miRNAs (UL22A-5p, US25-1-5p, UL22A-3p, US5-2-3p, UL112-3p, US25-2-3p, US25-2-5p, US33-3p, US5-1, UL36-5p, US4-5p, UL36-3p, UL70-5p and US25-1-3p), HCMV immediate-early mRNA and long non-coding RNA2.7, moreover, two host-encoded miRNAs (hsa-miR-218-5p and hsa-miR-21-5p) and beta-2-microglobulin RNA. UV-irradiated virions and DBs delivered viral miRNAs (US25-1-5p and UL112-3p) to the host cells, and miR-US25-1-5p was functional in a luciferase reporter assay. We conclude that virions and DBs carry miRNAs that are biologically functional and can be delivered to cells, which may affect cellular processes.

## Abbreviations

B2M, beta-2 microglobulin; DB, dense body; EBV, Epstein–Barr virus; HCMV, human cytomegalovirus; HSV, herpes simplex virus; IE, immediate-early; KSHV, Kaposi’s sarcoma-associated virus; lncRNA, long non-coding RNA; miRNA, microRNA; p.i., post-infection; RIN, RNA integrity number; UTR, untranslated region.

## Introduction

The prevalence of human cytomegalovirus (HCMV) ranges from 40 to 100 % worldwide [1]. A primary HCMV infection results in a lifelong latent or persistent infection. In newborns, HCMV may cause congenital infection and malformations [2]. In healthy people, HCMV infection is often asymptomatic, but in immunosuppressed individuals, it can cause life-threatening disease [3].

HCMV encodes at least four long non-coding RNAs (lncRNAs) [4] and 26 small non-coding mature microRNAs (miRNAs) (www.mirbase.org, Release-21) [5–8]. HCMV miRNAs are expressed during the lytic as well as the latent phase of infection [9] and are identified to target both host [10, 11] and viral mRNA transcripts [12, 13] and thereby regulate protein expression. They modulate various biological functions and are believed to mediate latency, viral replication, cell cycle, vesicle trafficking, virus assembly and immune evasion strategies [14, 15]. Furthermore, HCMV infection is also known to alter the cellular miRNA expression profile during the lytic and latent phases of infections [16–18].

Along with infectious mature HCMV particles (virions), a lytic infection produces defective viral particles such as dense bodies (DBs) and non-infectious enveloped particles [19–21]. These particles lack a mature capsid containing intact infectious viral DNA and are considered non-infectious. Virions are 200–300 nm in diameter and consist of a core capsid containing dsDNA. The capsid protects the viral genome and delivers it to the target cell nucleus, where it replicates and produces new virions. The capsid is covered with a tegument layer containing structural proteins and is further surrounded by a lipid bilayer containing viral glycoproteins [22].

During the virion assembly process, viral and cellular proteins [20, 23, 24] and mRNA and non-coding RNAs [25–29] are packaged into mature and defective viral particles. Upon *de novo* infection, these particles can deliver viral proteins to target cells and may affect cellular functions [30, 31]. While proteins have been shown to be encoded by particle-delivered mRNA, their functional role has not been tested so far [28, 32]. However, it is unknown whether host or viral encoded miRNAs are incorporated into virions and DBs, and if so, whether upon delivery of these miRNAs they are functional and could play a role in the early phase of virus infection.

In this study, we determined whether HCMV virions and DBs contain miRNAs. In proof-of-principle experiments, we examined whether these miRNAs are delivered to target cells and remain functional after delivery.

## Results

### Characterization of virions and DBs

**Fig. 1.**
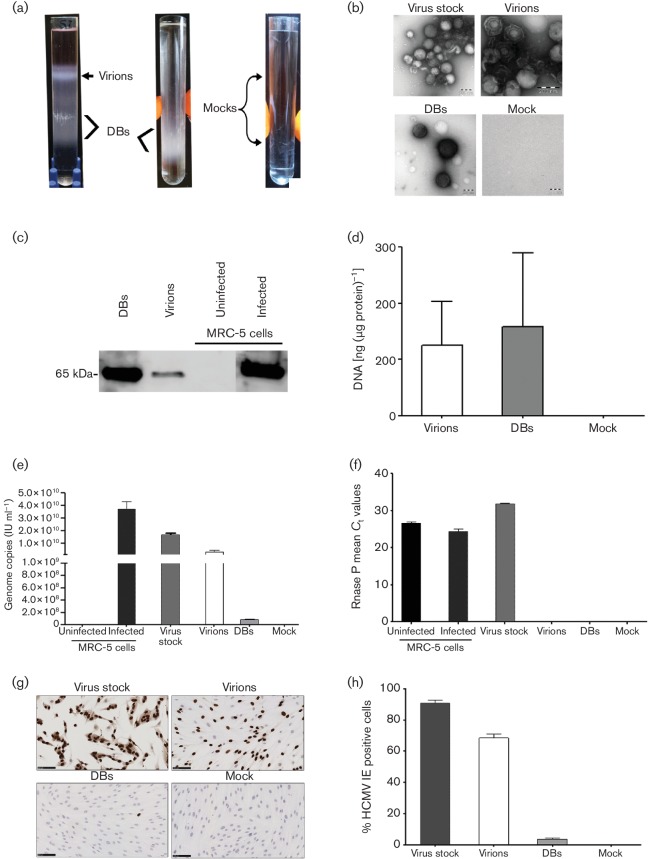
Isolation and characterization of virions and dense bodies (DBs). Media from MRC-5 cells infected with HCMV strain AD169 was collected and concentrated to pellets (viral stock), which were treated with RNase-ONE, and the virions and DBs were then purified by using potassium tartrate–glycerol gradient ultracentrifugation. (a) Representative tubes after gradient ultracentrifugation. (Left) Light-scattering upper band containing virions, lower band containing DBs. (Middle) A second ultracentrifugation was performed to further purify the DBs. (Right) No bands were seen in the mock samples. (b) Negative-staining electron microscopy showed virions (in upper band of gradient tubes), DBs (in lower band of gradient tubes), and some cellular debris in the viral stock; no particles were found in the mock samples. (c) Representative Western blot of lysed proteins from purified virions and DBs identified the HCMV structural protein pp65. Infected cells (5 days p.i., m.o.i. 0.1) and uninfected cells were used as positive and negative controls, respectively. (d) Concentration of DNA isolated from virions and DBs. (e) The number of HCMV genome copies [determined with World Health Organization (WHO) standards and gB Taqman] in the viral stocks, virions, DBs and mock samples; uninfected and infected MRC-5 cells were used as assay controls. (f) RNase P of host genome was found in the viral stock but not in the virions or DBs, confirming the purification of the virions and DBs; infected and uninfected MRC-5 cells were used as assay controls. (g) MRC-5 cells were infected with purified virions, DBs, virus stock and mock samples for 1 day, and analysed by immunocytochemistry. Brown, HCMV IE proteins; blue, nuclei; cells infected with viral stock served as a positive control. (h) Quantification of HCMV IE protein-positive cells. Values are mean±sem of three experiments performed in triplicate.

A summary of workflow and materials are graphically illustrated in (Fig. S1a). Ultracentrifugation of viral stocks resulted in two distinct light-scattering bands in the upper and lower parts of the gradient tubes; no band was observed in the mock samples containing no virus (Fig. 1a). Negative-staining electron microscopy revealed virions in the upper band and DBs in the lower band; no particles were found in mock samples. The viral stock contained virions, DBs and some cellular debris. Capsids were present in virions but not in DBs (Fig. 1b). The HCMV structural protein pp65 was detected in both purified virions and DBs by Western blot analysis. Uninfected and day 5 post-infected (p.i.) (m.o.i. 0.1) cell lysates served as negative and positive controls, respectively (Fig. 1c).

Prior to DNA isolation from purified virions, DBs and mock samples were treated with micrococcal nuclease to remove nucleic acids that were not within the particles. The mean DNA concentration [ng (µg protein)^−1^] was 125 in virions and 158 in DBs (Fig. 1d), and the purity ratios [optical density (OD) at 260/280 nm] were 1.6 and 1.3, respectively (Fig. S1b, available in the online Supplementary Material). No DNA or proteins were found in the mock sample. The number of HCMV genome copies (determined with WHO standards as IU ml^−1^) were 1.36×10^9^ in virions, 1.3×10^8^ in DBs and 3.74×10^10^ in infected cells (positive control); no copies were found in negative controls (Fig. 1e). The host genome (RNase P gene) was undetectable in the purified virions, DBs and mock samples. Suggesting that there was no residual host genome contamination, but it was found in viral stock (mean *C*_t_ 31.8), uninfected MRC-5 cells (mean *C*_t_ 26.6) and infected MRC-5 cells (mean *C*_t_ 24.3) (Fig. 1f).

To investigate the infectivity, the purified virions, DBs, viral stocks and mocks samples were added on MRC-5 cell monolayers, at 1 day p.i. fixed and analysed by immunocytochemistry with antibodies against HCMV immediate-early (IE) protein (Fig. 1g). The virions, DBs and viral stocks infected 68.4, 3.8 and 90.5 % of the cells, respectively; no cells were infected by the mock samples (Fig. 1g, h). The mean protein concentration was lower in purified virions than in DBs (95 versus 65 µg ml^−1^). The very low infection rates for DBs were probably caused by contaminating virions, which were present but very rarely detected by electron microscopy (data not shown). Thus, the virions and DBs were distinguishable by their density and shape, and both contained fairly similar amounts of DNA, although only virions resulted in a higher infection rate in MRC-5 cells.

### mRNA and lncRNAs are incorporated into virions and DBs

**Fig. 2.**
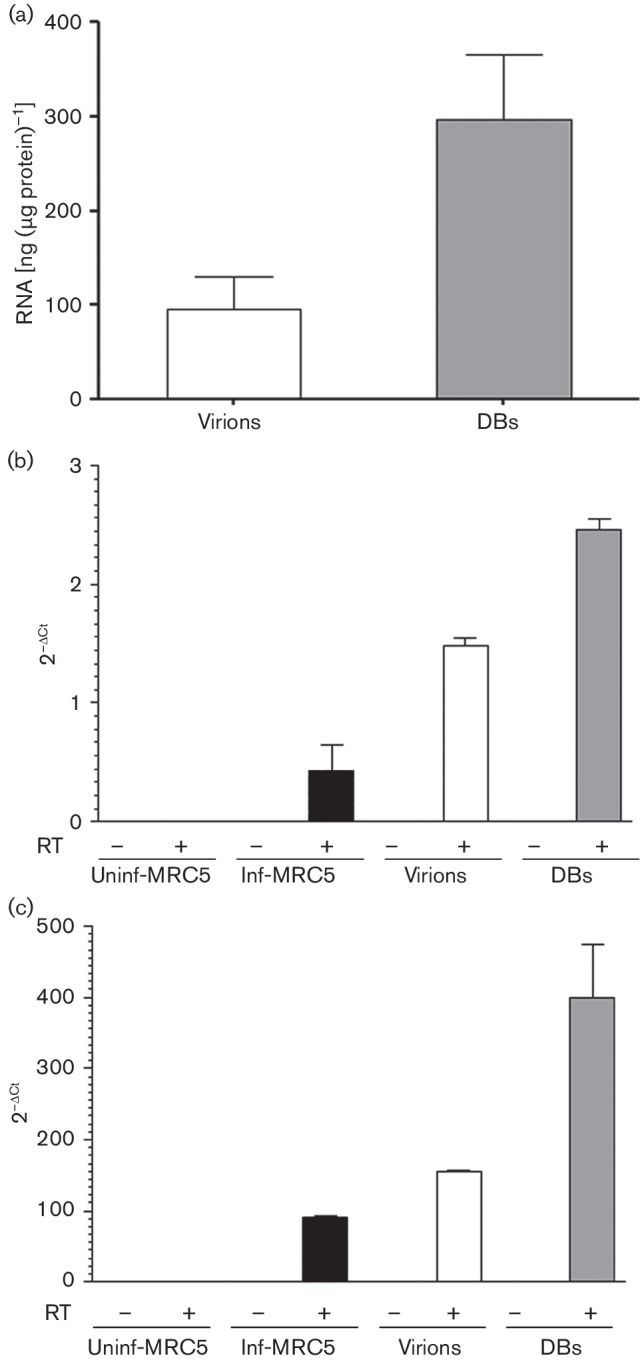
Analysis of RNA isolated from purified virions and dense bodies (DBs). Virions and DBs were purified from RNase-ONE-treated virus stocks, dialysed and treated with micrococcal nuclease; RNA was extracted, and during the RNA extraction the column was treated with DNase-I. (a) RNA concentrations of virions and DBs. Presence of (b) viral IE RNA transcripts and (c) lncRNA2.7 transcripts determined by TaqMan PCR assays. Values are mean±sem of three experiments performed in triplicate. RT, reverse transcriptase.

RNA molecules were present in the purified virions and DBs. The mean RNA concentration in the DBs and virions was 297 and 96 ng (µg protein)^–1^, respectively (Fig. 2a); the purity ratio (OD ratio at 260/280 nm) was 1.7 in both preparations (Fig. S1c). The cDNAs prepared from RNA samples of virions and DBs with reverse transcriptase were positive for HCMV IE mRNA (mean *C*_t_ values: 24.7 and 30.1, respectively; Fig. S2a), lncRNA2.7 (mean *C*_t_ values: 18.0 and 22.8, respectively; Fig. S2b) and host mRNA beta-2 microglobulin (B2M) (mean *C*_t_ values: 25.4 and 31.4, respectively; Fig. S2c), as determined by TaqMan PCR analysis. IE mRNA and lncRNA2.7 were present at 5 days p.i. in HCMV-infected cells (mean *C*_t_ values: 20.2 and 12.5, respectively) but not in uninfected cells. All Taqman RT-PCR assays in which reverse transcriptase was not added during cDNA preparation were negative (*C*_t_>35), suggesting that the TaqMan PCR amplification signal is specific for cDNAs and does not amplify residual DNA. Among the investigated RNAs, lncRNA2.7 was the most abundant transcript in virions, DBs and infected cells (5 day p.i.; m.o.i. 0.1) (Fig. 2b, c).

### Small RNAs and miRNAs are incorporated into virions and DBs

**Fig. 3.**
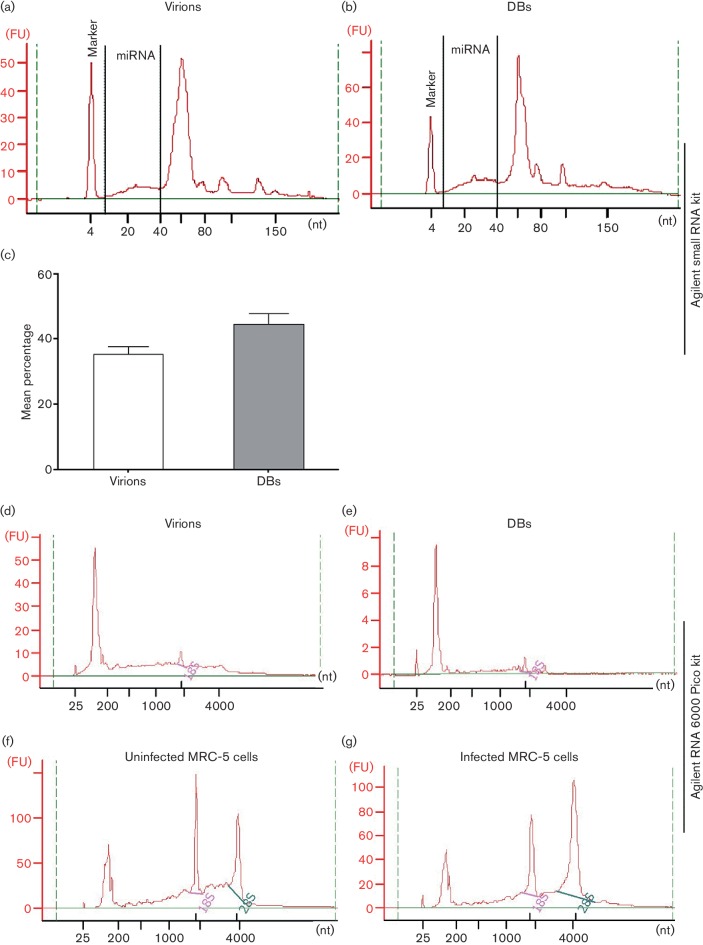
Bioanalyser-based analysis of RNA isolated from virions and dense bodies (DBs). To examine the small RNA species in virions (a) and DBs (b), a small RNA analysis kit (Agilent) was used. Representative electropherograms of RNA from virions and DBs show both miRNAs and small RNAs. (c) Mean percentages of miRNAs in virions and DBs; values are mean±sem of three experiments performed in triplicate. To inspect the total RNA species in virions (d) and DBs (e), an Agilent RNA 6000 Pico kit was used. Representative electropherograms of RNA from virions (d) and DBs (e) show very little 18S and almost no 28S rRNA, in contrast to the uninfected (f) and infected (g) MRC-5 cells. FU, fluorescence units; nt, number of nucleotides. Representative data from three experiments performed in triplicate are shown.

We found small RNAs, including miRNAs, in virions and DBs. To identify small RNA species and determine their length (6–150 nt), we used an Agilent Bioanalyzer with a small RNA kit. The electropherogram showed RNA molecules of various lengths in virions and DBs; a distinct peak of ~60 nt RNA had the highest fluorescence intensity among the total RNA species (Fig. 3a, b). miRNAs were also found in both DBs and virions (Fig. 3a, b). miRNAs accounted for 33.4 and 42.4 % of small RNAs in virions and DBs, respectively (data from three experiments) (Fig. 3c). Agilent RNA 6000 Pico kits (range, 25–6000 nt) were used to assess the total RNA species, and determine their length and integrity. This analysis revealed RNA molecules of various lengths, but much less 18S and almost no 28S rRNA, in virions and DBs (Fig. 3d, e). The RNA integrity number (RIN) was <3 in both virions and DBs. Analysis of the total RNA from uninfected and HCMV-infected cells collected at 5 days p.i. (m.o.i. 0.1) revealed two typical peaks of 18S and 28S rRNAs with the highest fluorescence intensity; the RINs were 7–9 (Fig. 3f, g).

Next, we used TaqMan RT-PCR assays to examine the presence of two host cell miRNAs and 15 HCMV-encoded miRNAs in the virions and DBs; we also performed cDNA reactions without reverse transcriptase to control for the residual viral DNA (Table 1). When reverse transcriptase was left out, no amplification signal was obtained (*C*_t_>35) from any of the miRNA samples prepared from the virions, DBs, uninfected cells or infected cells, demonstrating that the signal is not due to residual viral DNA (Figs S2a, b, 4 and 5). However, in uninfected cells in the presence of reverse transcriptase, amplification signal was obtained for one viral miRNA, US33-5p (mean *C*_t_ 33.8); therefore, this assay was considered non-specific (Fig. S3b, c).

**Fig. 4.**
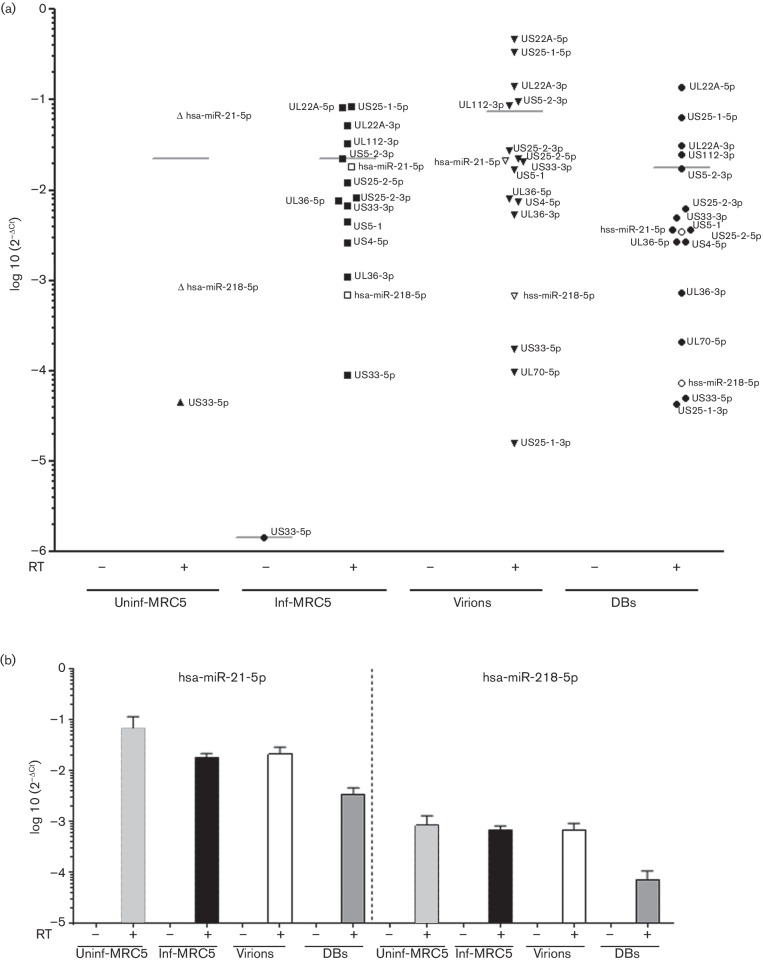
HCMV- and host-cell-encoded miRNAs in virions and dense bodies (DBs). To detect the miRNAs present in the virions and DBs, miRNA specific cDNA was synthesised in the presence or absence of reverse transcriptase enzyme (RT), and TaqMan PCR was performed. (a) TaqMan PCR analysis of 15 HCMV-encoded miRNAs and two host miRNAs in virions, DBs, and uninfected and infected MRC-5 cells. (b) Two host encoded miRNAs detected in virions, DBs, infected and uninfected control MRC-5 cells. The data were analysed with the 2^−ΔCt^ method, where Δ*C*_t_=miRNA of interest *C*_t_ value – Cel-miR39-3p spiked-in control *C*_t_ value. Values are mean±sem of three experiments performed in triplicate.

**Fig. 5.**
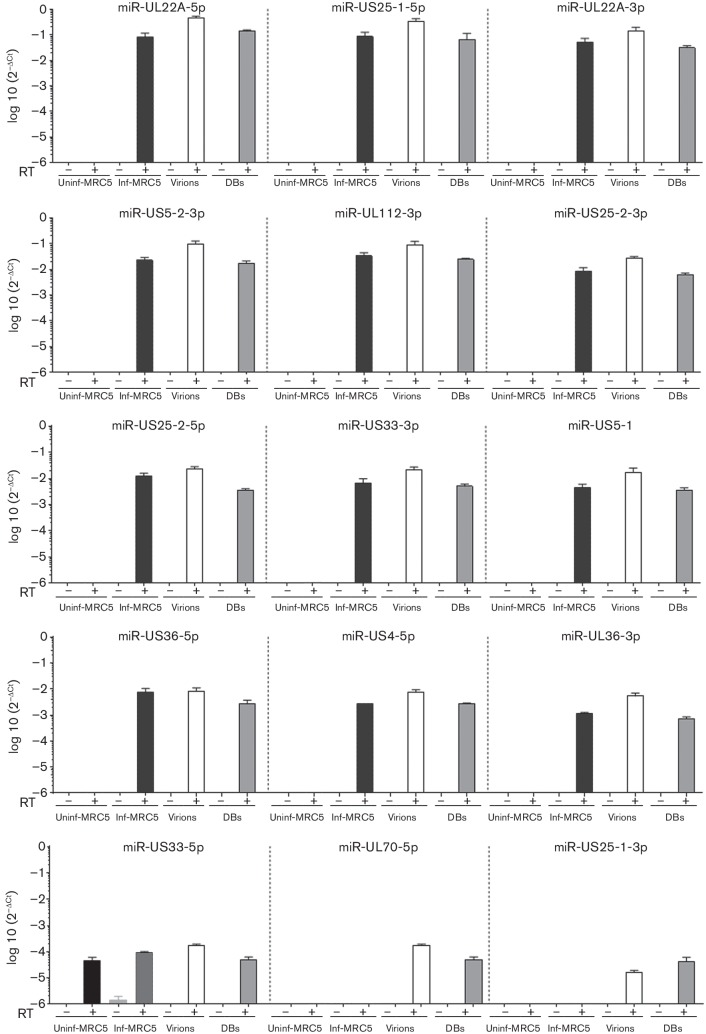
HCMV encoded miRNAs in virions, dense bodies (DBs), and infected and uninfected MRC-5 cells. To detect the miRNAs present in the virions and DBs, miRNA-specific cDNA was synthesised in the presence or absence of reverse transcriptase enzyme (RT), and TaqMan PCR was performed. TaqMan PCR analysis of 15 HCMV-encoded miRNAs, organised from the most abundant (top left) to the least abundant (bottom right), in virions, DBs, and uninfected and infected MRC-5 cells. The data were analysed with the 2^−ΔCt^ method, where Δ*C*_t_=miRNA of interest *C*_t_ value – Cel-miR39-3p spiked-in control *C*_t_ value. Values are mean±sem of three experiments performed in triplicate.

**Table 1.** TaqMan miRNA assays from Life Technologies

Count		Assay ID		Assay name	Mature miRNA sequence	miRBase ID*	miRBase accession no.
HCMV†					
1		6621		hcmv-miR-ul112	AAGUGACGGUGAGAUCCAGGCU	hcmv-miR-UL112-3p	MIMAT0001577
2		7677		hcmv-miR-ul22a	UAACUAGCCUUCCCGUGAGA	hcmv-miR-UL22A-5p	MIMAT0001574
3		6040		hcmv-miR-ul22a*	UCACCAGAAUGCUAGUUUGUAG	hcmv-miR-UL22A-3p	MIMAT0001575
4		197212_mat		hcmv-miR-ul36	UCGUUGAAGACACCUGGAAAGA	hcmv-miR-UL36-5p	MIMAT0001576
5		6481		hcmv-miR-ul36*	UUUCCAGGUGUUUUCAACGUGC	hcmv-miR-UL36-3p	MIMAT0004754
6		003183_mat		hcmv-miR-UL70-5p	UGCGUCUCGGCCUCGUCCAGA	hcmv-miR-UL70-5p	MIMAT0003342
7		197211_mat		hcmv-miR-us25-1	AACCGCUCAGUGGCUCGGACC	hcmv-miR-US25-1-5p	MIMAT0001581
8		8474		hcmv-miR-us25-1*	UCCGAACGCUAGGUCGGUUCUC	hcmv-miR-US25-1-3p	MIMAT0004755
9		5400		hcmv-miR-us25-2-3p	AUCCACUUGGAGAGCUCCCGCGG	hcmv-miR-US25-2-3p	MIMAT0001583
10		197201_mat		hcmv-miR-us25-2-5p	AGCGGUCUGUUCAGGUGGAUGA	hcmv-miR-US25-2-5p	MIMAT0001582
11		197197_mat		hcmv-miR-us33-3p	UCACGGUCCGAGCACAUCCA	hcmv-miR-US33-3p	MIMAT0004756
12		197227_mat		hcmv-miR-us33-5p	GAUUGUGCCCGGACCGUGGGCG	hcmv-miR-US33-5p	MIMAT0001584
13		197236_mat		hcmv-miR-us4	CGACAUGGACGUGCAGGGGGAU	hcmv-miR-US4-5p	MIMAT0003341
14		004641_mat		hcmv-miR-us5-1	UGACAAGCCUGACGAGAGCGU	hcmv-miR-US5-1	MIMAT0001579
15		6067		hcmv-miR-us5-2	UUAUGAUAGGUGUGACGAUGUC	hcmv-miR-US5-2-3p	MIMAT0001580
Human							
16		397		hsa-miR-21	UAGCUUAUCAGACUGAUGUUGA	hsa-miR-21-5p	MIMAT0000076
17		521		hsa-miR-218	UUGUGCUUGAUCUAACCAUGU	hsa-miR-218-5p	MIMAT0000275
18		1006		RNU48	GATGACCCCAGGTAACTCTGAGTG	na	na
					TGTCGCTGATGCCATCACCGCAGCGCTCTGACC		
*Caenorhabditis elegans*							
19		200		cel-miR-39	UCACCGGGUGUAAAUCAGCUUG	cel-miR-39-3p	MIMAT0000010

na, Not applicable.

*We updated the nomenclature based on the miRBase release-21; all the other information was obtained from the Life Technologies website.

†These were the only HCMV TaqMan miRNA assays available from Life Technologies when the study was initiated.

We found two host cell miRNAs (hsa-miR-218-5p and hsa-miR-21-5p) in the virions and DBs; both of these miRNAs were less abundant in infected cells than in uninfected cells. In all samples, hsa-miR-21-5p levels were higher than hsa-miR-218-5p levels (Figs S3c and 4b).

Moreover, we detected 14 HCMV encoded miRNAs (UL22A-5p, US25-1-5p, UL22A-3p, US5-2-3p, UL112-3p, US25-2-3p, US25-2-5p, US33-3p, US5-1, UL36-5p, US4-5p, UL36-3p, UL70-5p and US25-1-3p) in the virions and DBs, and 12 HCMV miRNAs (UL22A-5p, US25-1-5p, UL22A-3p, US5-2-3p, UL112-3p, US25-2-3p, US25-2-5p, US33-3p, US5-1, UL36-5p, US4-5p and UL36-3p) in the HCMV-infected cells at 5 days p.i. Overall, the abundance of the investigated miRNAs was similar in the virions, DBs and HCMV-infected cells (m.o.i. 0.1) (Fig. S3a, b). Of all the HCMV-encoded miRNAs examined, miR-US25-1-5p was the most abundant in the HCMV-infected cells, virions and DBs (Figs 5 and S3b, c). The two least abundant miRNAs (UL70-5p and US25-1-3p) were detected in the virions and DBs, but not in the infected cells (Figs 5 and S3b, c).

### Transfer of viral miRNAs to host cells

**Fig. 6.**
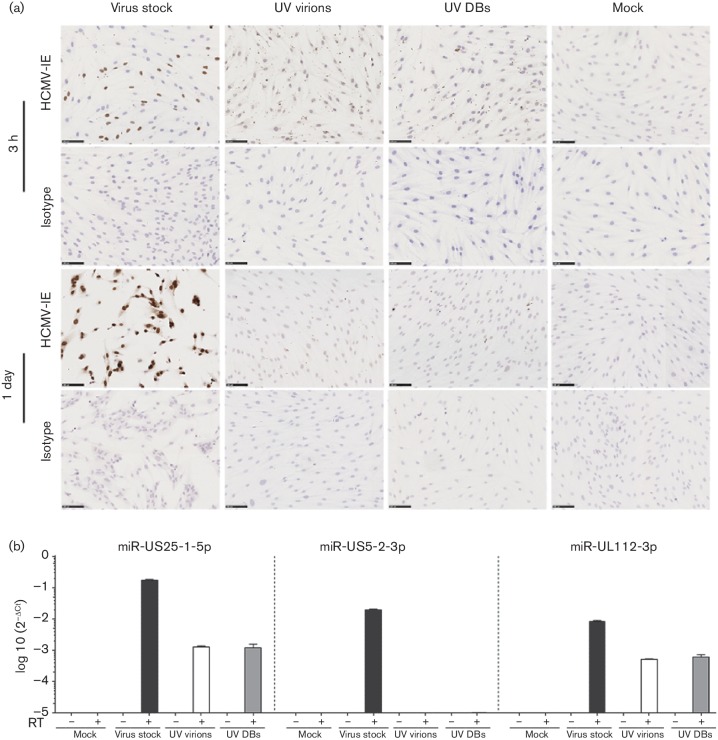
HCMV-encoded miRNAs from virions and DBs can be delivered to host cells. UV-irradiated virions, or DBs, or mock samples were added to MRC-5 cell monolayers; non-UV-treated virus stock was used as a positive control. (a) Immunocytochemistry staining for HCMV IE to validate the effectiveness of UV treatment of virions and DBs. UV-irradiated virions and DBs failed to infect the MRC-5 cells; non-UV-irradiated viral stocks resulted in infection of MRC-5 cells. Grey, HCMV IE staining; blue, haematoxylin staining of nuclei. (b) Two HCMV-encoded miRNAs, miR-US25-1-5p and miR-UL112-3p, were detected in cells treated with UV-irradiated virions and DBs. The data were analysed with the 2^−ΔCt^ method, where Δ*C*_t_=miRNA of interest *C*_t_ value – RNU48 *C*_t_ value. Values are mean±sem of three experiments performed in triplicate.

To determine whether virions and DBs deliver non-coding RNA and miRNAs to host cells, UV-inactivated virions and DBs were added to MRC-5 cell monolayers. None of the cells were infected; we did not detect IE RNA in cells at 3 h and 1 day after exposure (data not shown). However, a distinct cytoplasmic pattern of HCMV IE protein expression was observed at 3 h after addition of the particles using an HCMV IE-specific antibody (Fig. 6a). This pattern was not seen in uninfected cells, or 1 day after exposure of the cells to UV-irradiated virions or DBs, or when the typical IE nuclear staining pattern appeared in live HCMV-infected cells. We did not find lncRNA2.7 transcripts at 3 h (*C*_t_ values >35, data not shown) and 1 day post exposure (Fig. S3d). Of the three examined viral miRNAs, miR-US25-1-5p and miR-UL112-3p were present in cells exposed to UV-irradiated virions and DBs and cells infected with live viral stocks (Figs S3e and 6b); in all cases, miR-US25-1-5p was most abundant, but the difference was not statistically significant. No HCMV miRNAs were detected in mock-infected cells. In assays without reverse transcriptase, no amplification signal was detected, suggesting that the signal is specific to the cDNA and not due to residual viral DNA (Fig. 6a). These results indicate that both virions and DBs can deliver miRNAs to target cells in the absence of viral genome transcription or replication.

### Viral miRNA delivered to host cells is functional

**Fig. 7.**
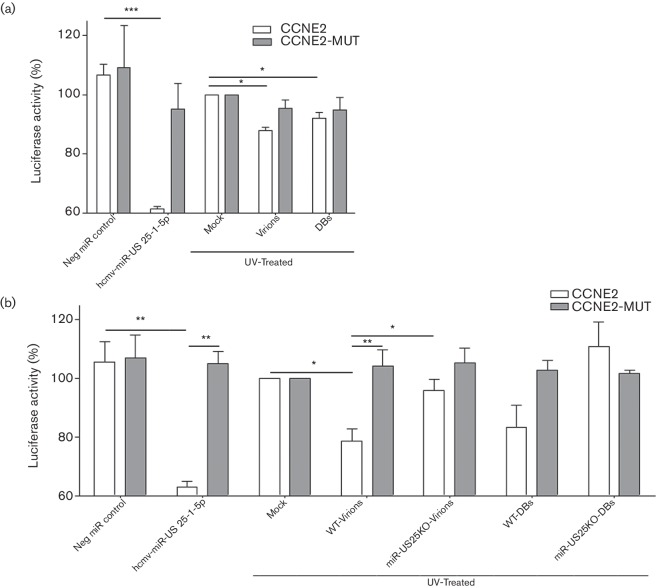
Virions and dense bodies (DBs) delivered miRNA to host cells may affect cellular functions, as shown by luciferase reporter assay. (a, b) HCMV-miR-US25-1-5p luciferase reporter assay – HEK-293 cells transfected with luciferase reporter constructs containing CCNE2 5′ untranslated region (CCNE2) or its mutated control (CCNE2-MUT) together with Renilla vector as internal control. (a) First luciferase experiment: on the transfected cells UV-irradiated virions, or DBs, or mock samples were added. (b) Second luciferase experiment: to further strengthen the data obtained from the first luciferase reporter assay experiments and improve the specificity of the assay. Virions and DBs were purified from the miR-US25 knockout (miR-US25KO) and its control wild-type (WT) virus stocks, and particles were UV irradiated and added to the HEK-293 cells transfected with CCNE2 or CCNE2-MUT luciferase reporters (the amount of particles was doubled in the second luciferase experiment compared to the first). In both the first and the second luciferase experiments as positive and negative miRNA controls hcmv-miR-US25-1-5p mimic or scramble miRNA mimics were co-transfected with the luciferase constructs, respectively. Cells were lysed 20 h later and luciferase activity was quantified. Firefly luciferase activity was normalized to Renilla luciferase; values are percentage mean±sem of three experiments performed in triplicate. *P*<0.05 was considered statistically significant. *P<0.05; **P<0.01; ***P<0.001.

To determine whether miRNAs delivered by virions and DBs are functional, we used a validated luciferase reporter assay designed for miR-US25-1-5p [33]. HEK-293 cells were transfected with 5′ untranslated region (UTR)-CCNE2-luciferase plasmid (CCNE2) or a mutated control plasmid (In the CCNE2-MUT plasmid miR-US25-1-5p seed sequence binding site in the 5′ UTR of the CCNE2 gene was replaced with *Eco*RI site). Later treated with UV-irradiated virions, or DBs, or mock samples; positive and negative controls were co-transfected with a miR-US25-1-5p mimic or scramble negative control, respectively. Luciferase activity was significantly decreased by 12 % in cells exposed to UV-irradiated virions (*P* *=*0.02) and by 8 % in cells exposed to UV-irradiated DBs (*P* *=*0.03) (Fig. 7a). In cells transfected with the mutated plasmid, luciferase levels decreased by 5 % after exposure to irradiated virions or DBs. Luciferase levels were also significantly lower (reduced by 39 %) in cells co-transfected with positive control miR-US25-1 mimic (*P* *=*0.005) as compared with cells transfected with scramble negative control (Fig. 7a).

To further strengthen the functionality of the delivered miR-US25-1-5p and also to dissect the potential non-specific downregulation of the luciferase levels in mutated plasmid, we used purified UV-treated virions and DBs from miR-US25KO and WT virus stocks. These particles were added to cells transfected with CCNE2 or CCNE2-MUT luciferase reporters (the amount of particles was doubled compared to the first luciferase experiment; (Fig.7a). WT-virions significantly lowered the CCNE2 luciferase levels (21 % reduced) compared with mock (*P* *=*0.04), or miR-US25KO control virions (*P* *=*0.04), while miR-US25KO virions did not significantly affect luciferase activity (4 % reduced compared with mock). Furthermore, in cells treated with WT-virions, significant downregulation was only observed in CCNE2 luciferase levels compared to CCNE2-MUT luciferase levels (*P* *=*0.01), and almost no change was noted in the levels of CCNE2-MUT luciferase. The CCNE2 luciferase levels were 17 % reduced with WT-DBs, and surprisingly 11 % increased with miRUS25KO DBs compared with the mocks, and none of these levels were statistically significant. CCNE2-MUT luciferase levels were almost unchanged and were not statistically significant compared with CCNE2 luciferase levels (Fig. 7b). The positive control miR-US25-1-5p mimic co-transfected cells had significantly lower CCNE2 luciferase levels compared with the scramble negative miRNA mimic controls (*P* *=*0.003) (Fig. 7b).

## Discussion

In this study, we found that purified virions and DBs contain HCMV-encoded IE mRNA, lncRNA2.7, at least 14 HCMV-encoded miRNAs, as well as host-encoded B2M mRNA and two host-encoded miRNAs (Table 1). We have provided evidence that virions and DBs can deliver viral miRNAs to target cells. In a proof-of-principle experiment, the delivered miR-US25-1-5p was functional. These observations suggest that particle-derived miRNAs may affect cellular functions early after entry of the virus before new miRNAs were produced.

The RNA molecules encoded by viruses and host cells are apparently present in particles of several viruses, such as HCMV [25, 27–29, 32, 34], Kaposi’s sarcoma-associated virus (KSHV) [26, 35], herpes simplex virus 1 (HSV-1) [36], adenoviruses [37] and Epstein–Barr virus (EBV) [38]. Thus, RNA carried by viral particles may play a role in viral biology.

In this study, we purified virions and DBs from the medium of infected cells. Gradient ultracentrifugation showed that DBs had a broader range of densities than virions. The virion and DB preparations were highly enriched, as demonstrated by the electron microscopy. However, it is difficult to produce DB preparations completely free of infectious virus and vice versa. In earlier studies, DB preparations were contaminated with virions [21, 27, 39] and were infectious [27]. The purity of our DB preparation was confirmed by the typical morphology of the particles by electron microscopy, the presence of pp65 protein and few copies of the HCMV genome, and the absence of the host cell genome (RNase P gene). Some infectivity remained after purification, which is probably mediated by contaminated virions. Another study suggested that DB preparations were non-infectious; after adding radioisotope-labelled thymine (hydrogen isotopes) and amino acids (carbon isotopes) to infected cells, the authors found no evidence of radiolabelled DNA in DB preparations. However, they also found no evidence of radioisotope labelled amino acids in the same DB preparations, which implied that no detectable DBs were present in that particular sample [40].

More recent studies have provided evidence that purified DBs contain various capsid proteins, which could be incorporated into the particles or be from contaminating virions in particle preparations. However, these studies did not search for viral DNA in DBs [23, 24].

Unexpectedly, we found similar amounts of DNA per microgram of protein in DB preparations and virions. No RNase P gene was found in the virions or DBs, suggesting little or no contamination with the host genome. The virions had more copies of the HCMV genome and were, as expected, much more infectious than the DBs, which only resulted in very limited infection that was probably caused by contaminating viral particles. These findings suggest that DBs do not deliver a functional and infectious HCMV genome to target cells. It was expected, as the viral DNA in DBs is not protected by a structural capsid for delivery to the nucleus in target cells. It is therefore not surprising that the DB preparations resulted in a very limited infection in MRC-5 cells. We speculate that the low infectivity of the DB preparations was from contaminating viral particles. Indeed, electron microscopy analyses rarely revealed virions in the DB preparations (data not shown).

### Virions and DBs incorporate various RNA species that may be functional

We used a Bioanalyzer that can distinguish various RNA species (e.g. rRNA, small RNA and miRNA) and their abundance. We found that HCMV virions and DBs contain small RNA species, including miRNAs. 18S and 28S rRNAs were most abundant in infected and uninfected cells but were found at very low concentrations in virions and DBs. We speculate that these cellular rRNAs are not available at the site of virus assembly in the cytoplasm to be incorporated into HCMV particles, as they are components of ribosomes and do not constitute free RNA molecules. In contrast, small RNA molecules are known to be abundant in HCMV-infected cells [41] and may thereby be available for incorporation into virions and DBs during their maturation, and they may be beneficial for the virus during the early phase of infection.

The functions of RNA transcripts in virions and defective particles are presently unclear. One study suggested that viral RNAs are selectively packed into HSV-1 virions [36]. RNAs ~70 nt long have been found in the virions of murine gammaherpesviruses 68 and 76, where they may be required for virion maturation and structural stability [42]. In murine leukaemia retrovirus, the genomic RNA molecule itself is thought to be a structural element of the particle [43]. In HCMV virions, RNA molecules may be incorporated non-specifically in proportion to the RNA concentration in the infected cells [28]. Alternatively, only subsets of RNA molecules may be incorporated into HCMV virions [32]. Viral RNA IRL4 (lncRNA2.7), IRL7 and UL21.5 have been found in HCMV virions and DBs [27, 32]. RNA molecules (300 and 500 nt long) were present in the capsid of HCMV particles. These RNA molecules can hybridise with the viral genomic DNA to form RNA–DNA hybrid structures that may have a role in viral DNA replication [44]. There is evidence that virions can deliver IRL4 (lncRNA2.7) and UL21.5 to the host cells [32]. Delivered UL21.5 RNA was translated into protein in the presence of the transcription inhibitor actinomycin-D. We found evidence of IE proteins in the cytoplasm of cells at 3 h post-treatment of UV inactivated virions and DBs (Fig. 6a); this cytoplasmic pattern of IE protein expression is frequently detected in tumours [45–47], but is not observed in *in vitro* infected cells. We speculate that it is translated from delivered IE mRNA or that IE proteins are delivered by the viral particles to produce this staining pattern. In theory, viral transcripts and proteins can be delivered by viral particles and regulate viral and host genes, affecting the cell cycle and viral latency, and preventing apoptosis immediately after delivery to cells and before new RNAs are made.

lncRNA2.7 is the most abundantly expressed early transcript in HCMV-infected cells [48], where it may help prevent apoptosis and maintain mitochondrial ATP production [49, 50]. Among the RNAs we assessed in the present study, lncRNA2.7 was the most abundant in virions, DBs and infected cells. However, UV-treated particles could not deliver the lncRNA2.7 transcripts to the cells (*C*_t_ values above 35). In contrast, one study found that RNA2.7 (IRL4) could be delivered to the cells from virus particles [32]. We also found evidence of HCMV IE transcripts and cellular mRNA B2M in both virions and DBs, confirming the results of two studies examining HCMV virions [25, 28]. However, in a previous study, the same group found no evidence of IE transcripts in HCMV virions [32]; this discrepancy was thought to reflect the use of different primers in their PCR assay.

### Functional relevance of mature miRNA in virions and DBs

We found HCMV miRNAs in both virions and DBs and provided proof of concept evidence that miRNAs can be delivered to host cells and be functional. We found that CCNE2 luciferase activity was significantly reduced using the WT virions; the mutated CCNE2 luciferase levels were not lowered. In sharp contrast, virions and DBs produced from the miR-US25KO virions did not lower the luciferase levels in either CCNE2 or mutated CCNE2 luciferase assays. These results suggest that this luciferase assay is specific to the delivered miR-US25-1-5p, as it was not affected by other delivered miRNAs or proteins. We also found that WT-DBs reduced the CCNE2 luciferase levels, but the relative change was not significant compared with miR-25KO DBs. Many cellular miRNAs are known to play major roles in viral biology. hsa-miR-122 is a cellular miRNA that is abundant in the liver and aids in replication of hepatitis C viral RNA [51, 52]. HCMV infection downregulates the hsa-miR-21-5p and over-expression of hsa-miR-21–5p inhibits the viral gene expression and replication [53, 54]. hsa-miR-21-5p is associated with many cancer types and has been found in KSHV virions [26]. We detected two host miRNAs, hsa-miR-218-5p and hsa-miR-21-5p, in the virions and DBs; both tended to be less abundant in infected cells than in uninfected cells, although the difference was not statistically significant. There may be an advantage for the virus to incorporate different cellular miRNAs.

Many targets of the HCMV-encoded miRNAs have been validated with luciferase reporter assays, suggesting potential effects of these miRNAs on host cell functions [55]. HCMV-encoded miRNAs can target both host and viral mRNAs and control post-transcriptional regulation of gene expression. miR-UL112-3p can target the gene encoding MHC class I polypeptide-related sequence B (MICB), perhaps to protect the cells from recognition and killing by natural killer cells [56]. miR-UL112-3p also targets IE72, and it has been proposed that this miRNA may play a role in regulation of HCMV latency [57]. miR-UL112-3p, miR-US5-1 and miR-US5-2 target multiple components (VAMP3, RAB5C, RAB11A, SNAP23 and CDC42) of the host secretory pathways. This may interfere with cytokine release early in the viral life cycle, prevent activation of the immune system and prepare for virus assembly [10].

miR-US25-1-5p was the most abundantly expressed miRNA in infected cells, virions and DBs. In infected cells, this miRNA targets the 5′ UTRs of multiple cell-cycle-regulating genes such as CCNE2, H3F3B and TRIM28 [33]. Hence, the delivered miR-US25-1-5p may target these genes. Furthermore, delivered miR-UL112-3p can target MICB and together with miR-US5-2-3p and miR-US25-1-5p it may target multiple secretory pathways. These miRNAs could also provide immune evasion strategies, and such viral strategies are not unique to HCMV. Viral miRNAs have been found in the virions of herpes B virus [58], EBV [38] and KSHV [26]. Furthermore, KSHV particles delivered miRNAs that were detected in the target host cells, and luciferase reporter assays showed that these miRNAs could be functional. Similarly, we found that HCMV virions and DBs delivered miR-US25-1-5p was functional in target host cells, as assessed by a luciferase reporter assays. Their potential functional relevance *in vivo* will need further in-depth investigation.

In conclusion, this study shows that virions and DBs carry HCMV encoded miRNAs that can be delivered to host cells, where they may alter cellular protein expression and play biologically relevant roles, especially during the early phase of HCMV's life cycle.

## Methods

Summary of workflow and materials are graphically illustrated in (Fig. S1a).

### Purification of the HCMV stocks, virions and DBs

The virions and DBs were isolated as previously described [21, 40], with minor modifications. Briefly, human lung fibroblasts (MRC-5; ATCC) at ~60 % confluence were infected with HCMV strain AD169 at an m.o.i. of 0.1. The medium was collected 3, 5, 7, 9 and 11 days p.i. Cellular debris was removed by centrifugation twice at 1500 ***g*** for 10 min and the supernatants were pooled and ultracentrifuged at 61 000 ***g*** for 1 h in a Type 45 Ti rotor (Beckman). To make a viral stock, the pellet was collected and suspended in 1× RNase-ONE reaction buffer, mixed for 30 min and treated with RNase-ONE (700 U ml^−1^; Promega) for 1 h at 37 °C. A mock viral stock was made as a control. The RNase-ONE-treated virus stocks and mock samples were diluted to 3 ml with TN buffer (0.05 M Tris-HCl, pH 7.4, 0.10 M NaCl) and overlaid on a 9 ml potassium tartrate/glycerol gradient prepared in TN buffer, and then ultracentrifuged at 200 000 ***g*** for 35 min in a SW40 Ti rotor (Beckman). Virions were collected from the upper part of the tube and DBs from the lower part. The DBs were further purified by gradient ultracentrifugation overnight. No bands were observed in the mock tubes; however, samples were collected from the upper and lower part of the tubes (combined and labelled as ‘mock’). The collected virions, DBs and mock controls were dialysed against 50 mM Tris-HCl buffer (pH 7.9) or PBS overnight in a Slide-A-Lyzer G2 dialysis cassette with a 2 K molecular weight cut-off membrane (Thermo Scientific). Strain AD169 of miR-US25 knock-out (miR-US25KO) and its control wild-type (WT) virus were obtained (BAC-transformed *Escherichia coli* stabs; a kind gift from Dr Finn Grey, Roslin Institute, University of Edinburgh). MRC-5 cells were infected with WT or miR-US25KO virus stock, virions and DBs were purified, and used for the investigation of the functionality of the miR-US25-1 in the luciferase reporter assay (second experiment).

### Negative-staining electron microscopy

The upper and lower light scattering bands (3 µl) collected from the gradient tubes were fixed with 1 % neutral buffered formalin, allowed to attach to a glow-discharged carbon-coated copper grid for ~5 min and washed with distilled water for 10 s. Excess solution was removed with filter paper. The sample was stained with 1 % uranyl acetate for 5–10 s, excess solution was removed and the sample was allowed to dry. Grids were examined in a Tecnai 12 Spirit Bio-Twin transmission electron microscope (FEI) at 100 kV. Digital images were taken with a Veleta camera (Olympus Soft Imaging Solutions).

### Western blotting

The upper and lower bands were lysed with 5× Laemmli sample buffer for 15 min, boiled for 10 min at 90 °C and centrifuged at 12 000 ***g*** for 5 min. The lysate was separated on a 4–15 % precast gradient polyacrylamide gel (Bio-Rad). Proteins were transferred to an Immobilon PVDF membrane (Millipore) with a Trans-Blot SD semi-dry transfer cell (Bio-Rad), blocked with 5 % non-fat dry milk (Bio-Rad) in TBS containing 0.1 % Tween-20 for 2 h, and incubated with mouse anti-HCMV-pp65 antibody (Virusys; 1 : 1000) overnight at 4 °C. The next day, the membranes were washed three times with PBS (15 min per wash), incubated with IRDye 680RD goat anti-mouse IgG (LI-COR Biosciences) for 1 h and washed three times with PBS (15 min per wash). The membrane was scanned for infrared signals with an Odyssey CLx system (LI-COR Biosciences).

### DNA and RNA isolation from virions, DBs and cells

Dialysed virions, DBs and mock samples were treated with micrococcal nuclease (2000 Kunitz units ml^−1^; Thermo Scientific) in the presence of 5 mM calcium chloride at 37 °C for 2 h. DNA was isolated with the QIAamp MinElute Virus Spin kit (Qiagen) without carrier RNA. RNA was isolated with the miRNeasy mini kit (Qiagen), including the DNase-I treatment or isolated with Trizol-LS reagent (Life Technologies). The DNA and RNA were also isolated from infected cells at 5 days p.i. (m.o.i. 0.1) and, uninfected cells. Next, quantified with a NanoDrop 2000, the optical density (OD) ratioat 260/280 nm was used as indicator for purity.

### DNA TaqMan PCR

DNA isolated from purified viral stock, virions, DBs and mock samples was subjected to absolute quantification of HCMV genome copies using the first WHO international standards (NIBSC) and HCMV gB Taqman assay [46, 59]. RNase P gene TaqMan assays were used to investigate the detection of host cell DNA. Uninfected MRC-5 cells and HCMV-infected cells served as assay controls.

### Immunocytochemical staining

To investigate the infectivity of the purified particles and verify the efficacy of UV irradiation, immunocytochemical staining was performed. In infectivity experiments, MRC-5 monolayers in eight-chamber slides were infected with purified virions, DBs, viral stock or mock. In delivery experiments, UV-treated virions and DBs were used. In both experiments, cell monolayers were fixed at 3 h and 1 day p.i. with 4 % paraformaldehyde for 10 min, washed with PBS and permeabilized with 0.5 % Triton X-100. The cells were blocked with 3 % H_2_O_2_ for 15 min, washed a few times with PBS, blocked with Fc receptor blocker and protein blocker for 20 min each (Innovex), and probed with HCMV IE antibody (1 : 100; Argene) overnight. The next day, the cells were incubated with ImmPRESS HRP anti-mouse IgG polymer (produced in the horse; Vector laboratories) for 1 h. Slides were washed a few times with PBS developed 5 min with substrate DAB and counterstained with haematoxylin.

### Bioanalyzer analysis of RNA

The total RNA (3 ng) isolated from the purified virions and DBs was analysed with an Agilent 2100 Bioanalyzer and Small RNA kits and RNA 6000 Pico kits (Agilent Technologies).

### cDNA synthesis and TaqMan PCR

RNA (60 ng) from virions, DBs, uninfected MRC-5 cells and MRC-5 cells collected at 5 days p.i. (m.o.i. 0.1) was primed with random hexamers, and cDNA was synthesised with the SuperScript-III First-Strand Synthesis system (Life Technologies), with or without reverse transcriptase. cDNAs (4 µl) were used as templates to detect HCMV IE [60] (TaqMan probe spans at the junction of exon 2 and 3, specific to cDNA), lncRNA2.7 (forward: CACCGAGAGAGATGATCTTTTTGTTCT; reverse: GCATGCCCTGCACATCCT; probe: FAM-CAGCAGCCAAACAATC-NFQ), and also host mRNA B2M (Hs00984230_m1; Life Technologies) by TaqMan PCR assays. For the detection of the 15 HCMV, and two host encoded mature miRNAs primer-specific cDNAs (1 ng RNA per 7.5 µl reaction) were synthesised with TaqMan miRNA cDNA synthesis kits (Life Technologies), with or without reverse transcriptase (Table 1). *C. elegans* synthetic miRNA mimic (cel-miR-39-3p; Life Technologies) was added to cDNA master mix (10 µM per reaction) as a spiked-in internal control. In a 384-well plate, 6 µl of TaqMan PCRs was assembled in triplicate with 1 µl of cDNA template. In MRC-5 cells, for the detection miRNA transferred from UV-irradiated virions and DBs, 15 ng of RNAs was used per cDNA reaction.

Fold differences were calculated with the 2^−^^ΔCt^ method, where Δ*C*_t_=*C*_t_ of target miRNA – *C*_t_ of cel-miR-39–3p or RNU48 (transfer experiments). Taqman assays with *C*_t_values ≤35 were considered positive.

### Transfer of viral RNA to host cells

Purified virions, DBs and mock samples were irradiated five times (in 500 µl Eppendorf® Safe-Lock microcentrifuge tubesusing default crosslink option and 50 mm distance from lamps) with a UV Stratalinker-1800 (Stratagene) and added to cultures of MRC-5 cells grown overnight in 12-well plates; volumes were adjusted based on the protein concentrations. Controls were infected with non-UV-irradiated viral stocks (m.o.i. 2). After 20 h, the monolayers were washed twice with PBS, treated with trypsin at 37 °C for 10 min, washed three times with PBS and centrifuged. The pellet was lysed, and RNA was isolated. Three viral miRNAs (miR-US25-1-5p, miR-US5-2-3p and miR-UL112-3p) were assayed with TaqMan PCR (15 ng RNA per cDNA reaction); RNU48 was used as endogenous control. The fold difference was calculated with the 2^−ΔCt^ method, where Δ*C*_t_=target miRNA *C*_t_ – RNU48 *C*_t_.

### Luciferase reporter assay

First luciferase experiment: to assess the functionality of miR-US25-1-5p delivered by virions and DBs, a validated luciferase assay for miR-US25-1-5p was obtained as a gift from Dr Finn Grey (Roslin Institute, University of Edinburgh) [33]. HEK-293 cells (ATCC) were grown overnight in 96-well plates, transfected with 100 ng of pGL4 luciferase vector containing CCNE2 5′ UTR or a mutated control vector (the miR-US25-1-5p seed sequence binding site in 5′ UTR of the CCNE2 gene was replaced with *Eco*RI site). CCNE2 5′ UTR was cloned into the *Xho*I and *Not*I cut sites in the pGL4 vector (personal communication from Dr Finn Grey). As an internal control, 50 ng of Renilla pRL-TK vector (a gift from Dr Qiang Ruan, Virus Laboratory, Affiliated to Shengjing Hospital, China Medical University) was co-transfected with Lipofectamine 2000 (Life Technologies). As a positive control, synthetic miR-US25-1-5p mimic (10 nM; Life Technologies) and a scramble negative control miRNA mimic (10 nM; Qiagen) were co-transfected with luciferase constructs in HEK-293 cells. Four hours after transfection, the medium was changed, and 4 h later UV-irradiated virions, or DBs, or mock samples were added; volumes were adjusted based on the protein concentrations. After 20 h, the cells were lysed and the luciferase activity was quantified with the Dual-Luciferase Reporter Assay system (Promega); Firefly luciferase activity values were normalised to Renilla luciferase activity (internal control) moreover, converted to a mean percentage. Second luciferase experiment: by using the above protocol, to further strengthen the functionality of the delivered miR-US25-1-5p and also dissect the non-specific downregulation of the luciferase activity of the mutated plasmid; we performed luciferase assays using purified UV-treated virions and DBs from the miR-US25KO and WT virus stocks. In this second experiment, we added double the amount of particles compared to the first experiment.

### Statistical analysis

Luciferase levels were expressed as percentage mean±sem of three experiments (performed in triplicate) and analysed with a *t*-test using the Graph Pad Prism. *P* values <0.05 were considered statistically significant.

## Supplementary Data

920 Supplementary File 1
