# Supplementary material for: Human cytomegalovirus microRNAs are carried by virions and dense bodies and are delivered to target cells
Source: J Gen Virol. 2017 Jun 5;98(5):1058–72. doi: 10.1099/jgv.0.000736 (PMC5656795; doi:10.1099/jgv.0.000736)
Supplement: Supplementary File 1 [file jgv-98-1058-s001.pdf]

SUPPLEMENTAL FIGURE LEGENDS

Fig. S1. Characterization of virion and DBs (a) A brief flow chart of experiments. (b) Virions and DBs were purified from RNase-ONE treated virus stocks, dialyzed and treated with micrococcal nuclease; DNA was extracted and quantified, the purity of DNA is the estimated from the OD values obtained at 260/280 nm. OD, optical density. (c) RNA was isolated from purified virions and DBs, while during the RNA extraction columns were treated with Dnase-I. OD, optical density. (d) Mean Ct values of gB Taqman assays of viral stocks, virions, DBs, and mock samples (used during the WHO standards estimation of copy numbers); DNA from uninfected and infected MRC-5 cells were used as assay controls, analysed data is shown in Fig 1e. Values are mean ± SEM of three experiments performed in triplicate.

Fig. S2. Taqman PCR mean Ct values of various RNA transcripts found in HCMV virions and DBs. Virions and DBs were purified from RNase-ONE treated virus stocks, dialyzed and treated with micrococcal nuclease, and RNA was extracted; during the RNA extraction column was treated with Dnase-I. cDNA reactions were made with or without reverse transcriptase and TaqMan assays were performed. (a) Presence of viral IE RNA transcripts, (b) long noncoding RNA 2.7 transcripts, (c) cellular beta-2-microglobulin transcripts in virions and DBs. Values are mean ± SEM of three experiments performed in triplicate. RT, reverse transcriptase.

Fig. S3. miRNA taqman assays of RNA obtained from virions and DBs, and transfer of viral RNAs from particles to target host cells (a) Mean Ct values of host cell miRNAs, analysed data is shown in fig. 4b and (b) HCMV miRNAs in purified virions, DBs and mock-infected and HCMV infected cells analysed data is shown in fig. 5. (c) Fold change of HCMV miRNA levels in purified virions, DBs, and mock-infected and HCMV infected cells, analysed data is shown in fig. 5. (d) Transferred lncRNA 2.7 in cells treated with UV-irradiated virions or DBs or with viral stock or mock samples. (e) Mean Ct values of

transferred HCMV miRNAs; host RNU48 served as an endogenous control, analysed data is shown in fig. 6b. Values are mean ± SEM of three experiments performed in triplicate.

Figure S1

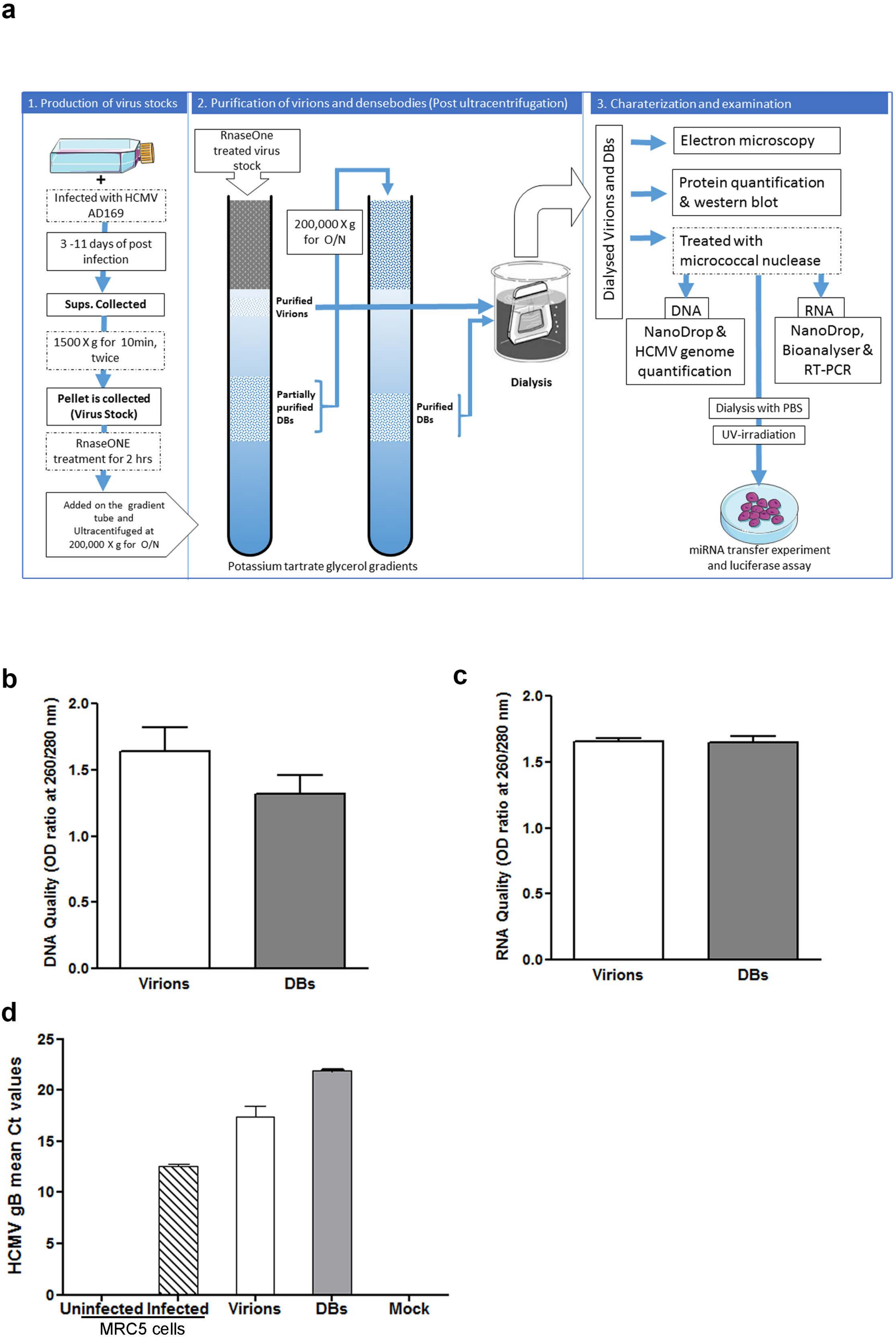

a
1. Production of virus stocks
+
Infected with HCMV AD169
3 -11 days of post infection
Sups. Collected
1500 X g for 10min, twice
Pellet is collected (Virus Stock)
RnaseONE treatment for 2 hrs
Added on the gradient tube and Ultracentifuged at 200,000 X g for O/N
2. Purification of virions and densebodies (Post ultracentrifugation)
RnaseOne treated virus stock
200,000 X g for O/N
Purified Virions
Partially purified DBs
Purified DBs
Dialysis
Potassium tartrate glycerol gradients
3. Charaterization and examination
Dialysed Virions and DBs
Electron microscopy
Protein quantification & western blot
Treated with micrococcal nuclease
DNA
NanoDrop & HCMV genome quantification
RNA
NanoDrop, Bioanalyser & RT-PCR
Dialysis with PBS
UV-irradiation
miRNA transfer experiment and luciferase assay
b
DNA Quality (OD ratio at 260/280 nm)
2.0
1.5
1.0
0.5
0.0
Virions
DBs
c
RNA Quality (OD ratio at 260/280 nm)
2.0
1.5
1.0
0.5
0.0
Virions
DBs
d
HCMV gB mean Ct values
25
20
15
10
5
0
Uninfected
Infected
MRC5 cells
Virions
DBs
Mock

Figure S2

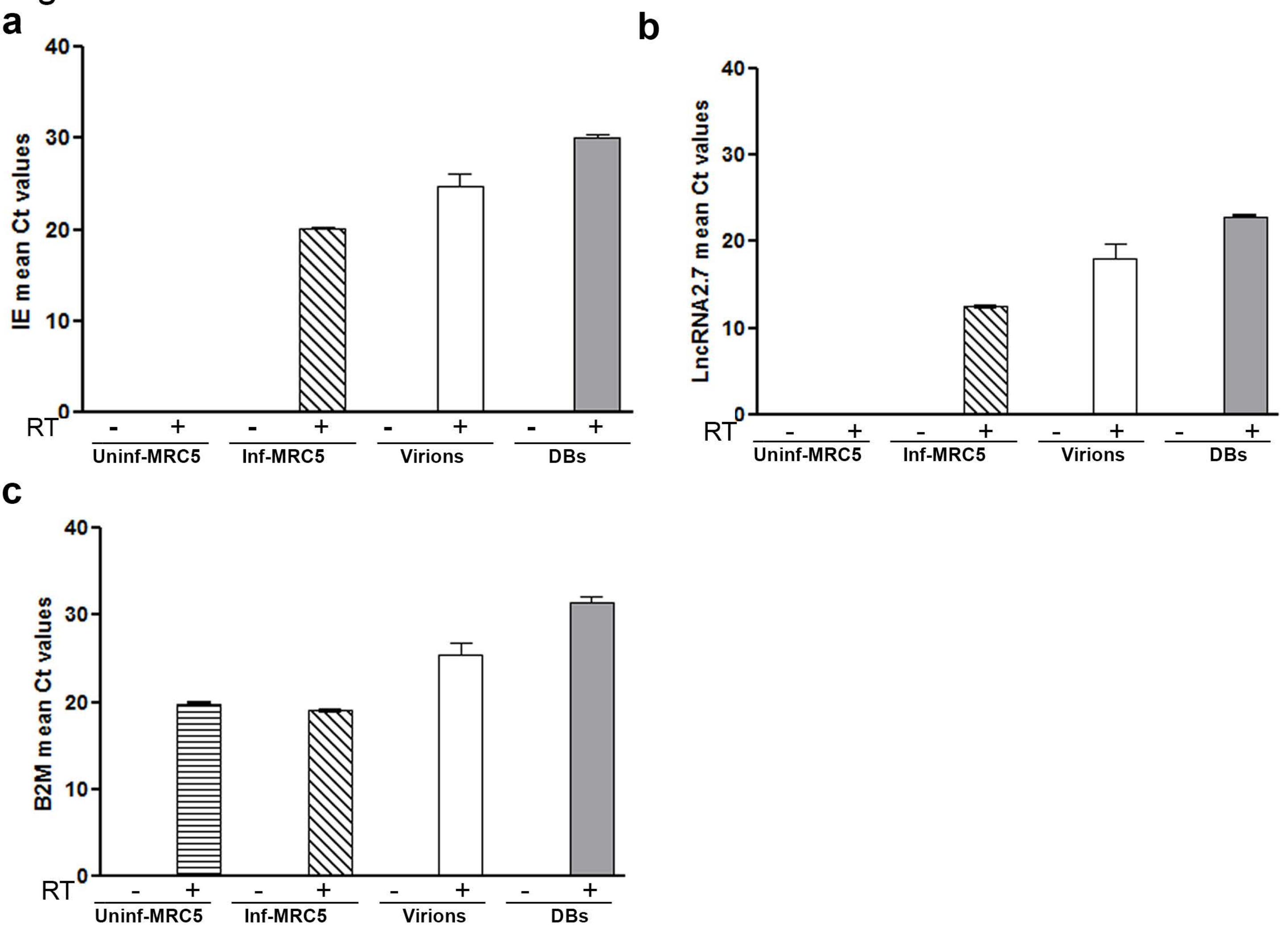
a
IE mean Ct values
40
30
20
10
0
RT
-
+
Uninf-MRC5
Inf-MRC5
Virions
DBs
b
LncRNA2.7 mean Ct values
c
B2M mean Ct values

Figure S3

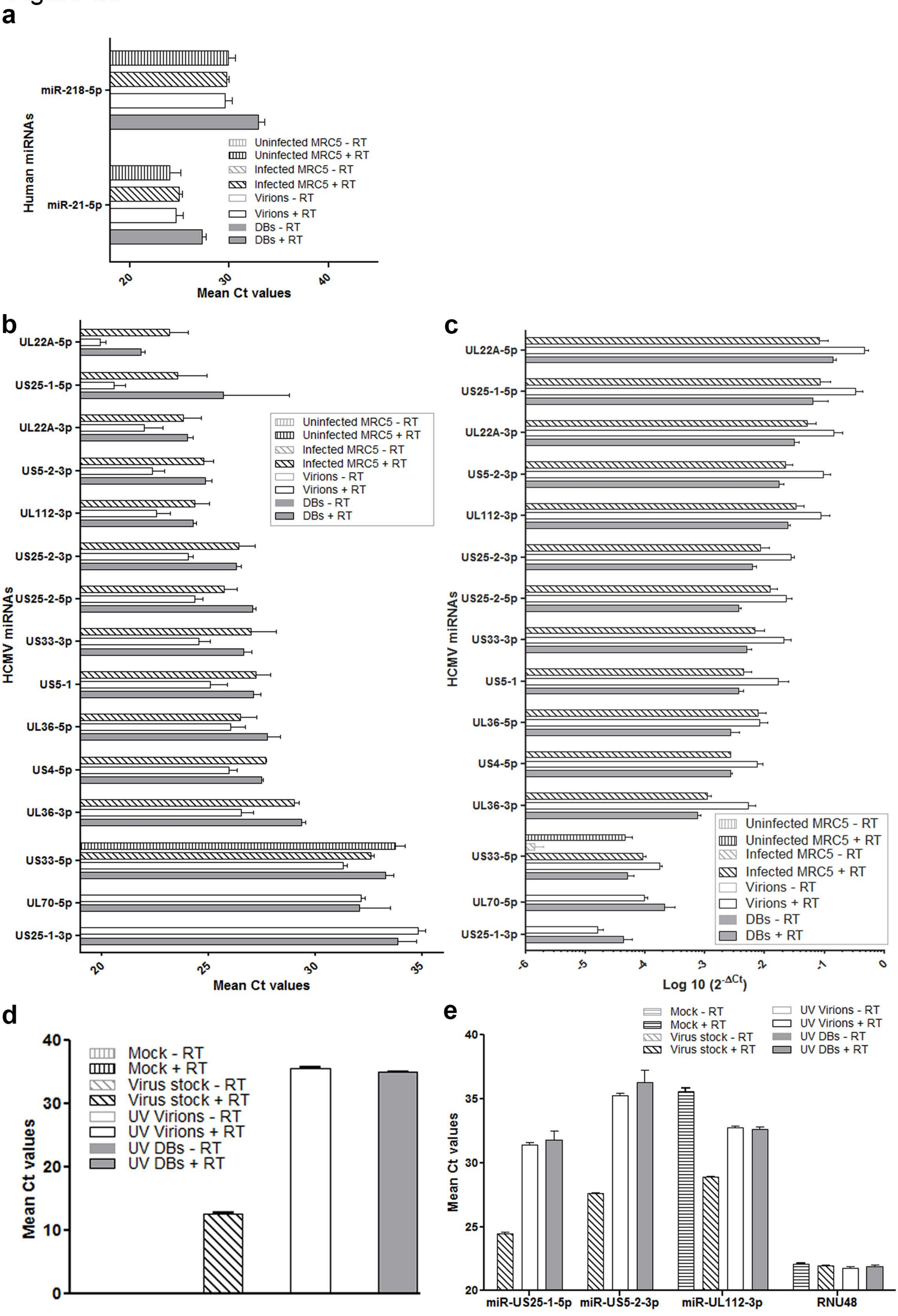

a
miR-218-5p
miR-21-5p
Human miRNAs
Uninfected MRC5 - RT
Uninfected MRC5 + RT
Infected MRC5 - RT
Infected MRC5 + RT
Virions - RT
Virions + RT
DBs - RT
DBs + RT
20
30
40
Mean Ct values
b
UL22A-5p
US25-1-5p
UL22A-3p
US5-2-3p
UL112-3p
US25-2-3p
US25-2-5p
US33-3p
US5-1
UL36-5p
US4-5p
UL36-3p
US33-5p
UL70-5p
US25-1-3p
HCMV miRNAs
20
25
30
35
Mean Ct values
c
Log 10 (2^-ΔCt)
-6
-5
-4
-3
-2
-1
0
d
Mean Ct values
Mock - RT
Mock + RT
Virus stock - RT
Virus stock + RT
UV Virions - RT
UV Virions + RT
UV DBs - RT
UV DBs + RT
0
10
20
30
40
e
miR-US25-1-5p
miR-US5-2-3p
miR-UL112-3p
RNU48
25
35
